# Hydrophilic ZnO thin films doped with ytterbium and europium oxide

**DOI:** 10.1038/s41598-022-14899-z

**Published:** 2022-07-05

**Authors:** Tomasz Tański, Marta Zaborowska, Paweł Jarka, Anna Woźniak

**Affiliations:** 1 Department of Engineering Materials and Biomaterials, Faculty of Mechanical Engineering, Silesian University of Technology, Konarskiego 18A, Gliwice, Poland; 2 Materials Research Laboratory, Faculty of Mechanical Engineering, Silesian University of Technology, Konarskiego 18A, Gliwice, Poland

**Keywords:** Optical materials and structures, Materials for optics, Nanoscale materials, Techniques and instrumentation

## Abstract

Hydrophilic photocatalytically active ZnO and ZnO thin films doped with Yb_2_O_3_ and Eu_2_O_3_ (rare earth metal oxide, REM) with optical transmittance exceeding 76% in the visible light range (λ = 550 nm) were prepared by a combination of sol–gel technique, spin-coating and high temperature thermal treatment at 500 and 600 °C. The thin films were tested using advanced research methods, i.e.: morphology and topography and fractures along with approximate thickness values were investigated on scanning electron microscope (SEM), chemical composition was determined using X-ray Energy Dispersive Spectroscopy (X-ray Energy Spectroscopy), topography and roughness were measured on atomic force microscope (AFM), water contact angle values were determined by sitting water droplet method, optical properties of the fabricated materials were investigated using UV/Vis spectrophotometer. The decolorization efficiency of rhodamine B in aqueous solution was analyzed over a period of 190 min, obtaining degradation rates of: 54.7% and 43.1%, for ZnO and ZnO coatings doped with ytterbium oxide and europium oxide, respectively. The roughness of thin hybrid coatings did not exceed 50 nm, ensuring effective absorption of electromagnetic radiation by the layers. The methodology presented by the authors for the fabrication of thin hybrid films characterized by the key properties of self-cleaning coatings can be successfully applied to coatings of photovoltaic panels and architectural glass structures.

## Introduction

Surface wettability is one of the most important phenomena occurring at the interface between solids, liquids and gases. Exploiting the knowledge of a liquid's ability to wet a given surface, first observed and described by Thomas Young in 1805, allows scientists around the world to design and fabricate controlled engineering materials for a wide range of applications^1^. Advances in engineering enable the fabrication of materials with different surface energies and morphologies, as well as smart materials with switchable wettability depending on conditions such as radiation or temperature using many methods such as 3D printing^2^, template-based synthesis methods^3^, phase separation^4^, spin-coating^5^, electrospinning^6^, and molecular self-organization^7^. For many phenomena used in the world of technology and science, inspiration comes from nature. An excellent example of the phenomenon of hydrophobicity can be seen in the flagship example of lotus leaves, which repel water droplets due to their micro- and nano-hierarchical surface morphology and low surface energy^8^. The hydrophobic properties that characterize the leaf surfaces of these plants have become a motivation for the fabrication of self-cleaning coatings that can find applications in everyday life, including protection of photovoltaic panels from dust and dirt^9,10^, anti-corrosion coatings^11^, anti-reflective coatings^12^, self-cleaning glass in architectural applications^13^, hydrophobic fabrics^14^ or flexible superhydrophobic surfaces^15,16^. The opposite of hydrophobicity is hydrophilicity, which is the ability of a liquid to wet a specific surface. Similarly to hydrophobic ability, the phenomenon of hydrophilicity is also used in the natural world by many plant species such as Ruellia devosiana or Calathea zebrina. The advantages of constant wetting of plant leaves include the absence of drought risk, the possibility of water and nutrient uptake in plants with undeveloped vascular systems for water transport, faster water evaporation by increasing the water–air interface, and insect capture by carnivorous plants^17^. Hydrophilic properties are particularly important for designed surfaces of windows, mirrors or shower enclosures, but also for easy-to-clean household goods, traffic signs or antifouling paints^18^. Another no less important application of surface hydrophilicity is photocatalytic self-cleaning surfaces, in which the cleaning process is based on the chemical decomposition of adsorbed dust and organic dirt.

Coatings that exhibit self-cleaning properties have attracted the attention of researchers because of their ability to clean surfaces without the use of an additional support agent. It becomes extremely important to understand the mechanism of surface wetting, which in this case is the key phenomenon responsible for the wide application potential of self-cleaning coatings. There are two mechanisms responsible for surface cleaning, i.e.: hydrophilic and hydrophobic. The factor conditioning one of the mechanisms of the phenomenon occurring on the coating surface is the value of the water contact angle (θ), which is defined as the angle between the wetted surface and the wetting liquid, most often water. The value of the water contact angle is influenced, among other things, by the surface roughness, morphology, but also by the chemical composition, which defines the surface energy. The division of surfaces according to the value of the water contact angle is as follows: hydrophilic if θ < 90°, superhydrophilic for θ < 10°, hydrophobic for water contact angle above 90°, and superhydrophobic for values θ ≥ 150°. The cleaning phenomenon of the coating in the hydrophilic mechanism is based on the spreading of water droplets across the surface and the removal of dirt and contaminants, while the hydrophobic mechanism is based on the sliding of water droplets across the surface of the coating and the cleaning of contaminants. Undoubtedly, an additional advantage of hydrophilic coatings with suitable solid-state metal oxide chemical compositions is the ability to chemically clean contaminants from the surface using light at specific wavelengths. Coatings with MOX (semiconductor metal oxide) chemical composition characterized by optical properties allow to perform photocatalysis process consisting in chemical decomposition of organic dirt and other contaminants present on the surface of the coating by absorption of sunlight. The hydrophilic properties of the coatings cause the formation of thin "water films" on the surface of the coatings, reducing the water contact angle and allowing dirt to be washed off. MOX thin films absorb light with energies equal to or higher than the energy band gap, producing excited charge carriers, i.e.: positively charged electron holes (h+) and negatively charged electrons (e−). Some of the photo-excited charges migrate to the surface of the MOX film where h+ oxidizes organic molecules while e− combines with atmospheric oxygen to form superoxide radicals capable of breaking down organic contaminants. The entire process results in cleaning of the coating surface by converting organic molecules into carbon dioxide and water molecules^19^.

Using knowledge of the wettability of different surfaces and mimicking the effects of nature, it is possible to produce cost-effective and uncomplicated self-cleaning coatings for advanced applications. Examples include coatings used on photovoltaic panels or architectural glass structures. Self-cleaning coatings use raindrops and sunlight in the cleaning process, eliminating the need for manual cleaning, thereby reducing maintenance costs for glass surfaces.

The most commonly used metal oxides that exhibit photocatalytic properties and suitable wetting properties for their thin film forms are, among others titanium dioxide (TiO_2_)^20–23^, zinc oxide (ZnO)^24–28^, tin oxide (SnO_2_)^29–32^, vanadium oxide (V_2_O_5_)^33–36^, and tungsten oxide (WO_3_)^37–41^. Among these materials, TiO_2_ is gaining the most popularity in self-cleaning coating applications due to its outstanding photocatalytic properties and superhydrophilicity. The other important oxide in self-cleaning coating applications is zinc oxide, which exhibits a similar chemical surface cleaning mechanism to TiO_2_ using photocatalysis. A noteworthy property of both materials is the superhydrophilicity obtained under ultraviolet irradiation, allowing for a more efficient cleaning process of surfaces to which ZnO or TiO_2_ coatings have been applied.

Mufti N. et al. obtained zinc oxide thin films with controlled thickness using the potential of sol–gel and spin-coating techniques, which were then subjected to a calcination process at 500 °C. By selecting appropriate rotational speed values of the spin-coating process, thin films with thicknesses in the range of 250–1050 nm were obtained, characterized by hydrophobic properties, which became superhydrophilic under solar irradiation, providing a suitable material for self-cleaning coatings. Moreover, the thickness of ZnO thin films did not significantly affect the optical properties, including the energy gap width of the materials, which was 3.2 eV^25^. In the work^42^ thin films were obtained in the process of magnetron RF sputtering, as a result of which thin ZnO layers were obtained with a roughness of 50.76 nm and optical transmittance above 80%, with a corresponding band gap width of 3.3 eV. Moreover, the authors investigated the influence of the applied ZnO coating on the silica substrate on the value of radiation reflection, which showed a decrease of the value from 12% in the wavelength range 400–1030 nm to approx. 2%, in particular for the wavelength of 600 nm. The favorable value of radiation reflection in the obtained ZnO thin layers allowed to increase the efficiency of the solar cell to which the coating was applied and the conversion of solar radiation energy into electric energy. The doping of the zinc oxide crystal lattice with lanthanide ions allows to reduce the fast recombination of charge carriers and thus to obtain better chemical purification efficiencies of coatings. Saif et al. in their work fabricated ZnO/Sm^3+^ thin films with varying values of molar concentration of samarium ions using a hydrothermal method obtaining highly photocatalytically active coatings. The doping of zinc oxide with lanthanide ions allowed the energy gap width to increase to a maximum value of 3.26 eV, consistent with the Moss-Burstein effect, thereby increasing the efficiency of the photocatalysis process and improving the self-cleaning properties of the coatings. In addition, the authors of this paper also investigated the effect of ZnO/Sm^3+^ coatings on the lethality of *S. aureus* bacterial colonies under ultraviolet radiation, which was almost 90%, compared to a value of about 11% in the dark without additional radiation ^43^. Another application of doping other ions in the crystal lattice structure of ZnO thin films is the ability to control the wetting properties of the resulting coatings. Nundy et al. showed that 6% concentration of hafnium (Hf) ions in zinc oxide thin films led to a reduction in the contact angle, while 15% concentration of hafnium in ZnO coatings caused an increase in the water contact angle value, thus increasing the roughness of the coatings and superhydrophobic properties. Apart from the significant self-cleaning properties of the obtained coatings, the authors also investigated the effect of Hf/ZnO thin films on antibacterial properties. Using the E. *coli* bacteriostatic ring experiment as an example, it was shown that all the obtained thin films exhibited distinctive antibacterial properties^44^. The effect of the morphology of ZnO thin films on the controlled change of wettability was investigated in the work^45^, in which the authors fabricated thin films with morphologies of nanowires, microflowers and porous microflowers. It was also proved that the control of the morphology of ZnO films and its effect on the water contact angle values and the obtained hydrophilic and superhydrophilic properties were independent of the application of ultraviolet radiation. The obtained ZnO thin films had transmittances above 80% and exhibited energy gap widths in the range of 3.1–3.6 eV. Moreover, the lowest value of the water contact angle was observed for the porous microflowers, which was associated with an increase in the surface roughness of the thin film and more free sites in the film microstructure, and this translated into a higher degree of wettability according to the Wenzel model. Despite the desirable hydrophilic properties of self-cleaning coatings, targeted surface modifications of ZnO thin films are used to obtain superhydrophobic properties that will clean the surface to which they are applied according to the hydrophobic mechanism. In the work^27^, octadecyltrichlorosilane modification was used to achieve superhydrophobic properties of ZnO thin films, which remained stable even after UV irradiation.

Based on the above, ZnO is one of the better candidates for developing self-cleaning coatings on the surfaces of photovoltaic panels or architectural glass structures, among others. The treatments used are aimed at modifying the roughness, morphology, water contact angle values, and thus improving the wetting and photocatalytic properties, which can significantly revolutionize the field of research on self-cleaning coatings. Yb_2_O_3_ and Eu_2_O_3_, which are rare earth oxides, are most commonly used to fabricate materials that find applications in optoelectronics and sensorics due to their distinctive optical properties^46–50^.

Based on the authors' current knowledge, no attempts have been made so far to fabricate hybrid ZnO thin films doped with Yb_2_O_3_ and Eu_2_O_3_ oxides for self-cleaning coating applications. Therefore, the aim of this work is to combine the potential of sol–gel and spin-coating techniques to obtain hydrophilic, photocatalytically active self-cleaning coatings and to investigate the influence of rare earth oxides on the roughness, wettability and optical properties of thin films, which can be applied as coatings for photovoltaic panels or coatings for glass structures.

## Materials and methods

The following were used to prepare solutions by the sol–gel technique: zinc nitrate dihydrate (ZnAc, Zn(CH_3_COO)_2_·2H_2_O, Sigma Aldrich, purity: 99.999%), ytterbium chloride hexahydrate (YbCl_3_·6H_2_O, Sigma Aldrich, purity: 99.9%), europium hexahydrate chloride (EuCl_3_·6H_2_O, Sigma Aldrich, purity: 99.9%), ethanol (EtOH, purity: 99.8%), N,N-dimethylformamide (DMF, purity: 99.8%), and polyvinylpyrrolidone (PVP, Sigma Aldrich, Mw = 1,300,000 g/mol). During the procedure, measured amounts of PVP polymer powder were mixed with ethanol to obtain a 10% wt. PVP/EtOH solution. In the next step, precursor solutions were prepared in DMF solvent, for which 1.2 g of zinc nitrate dihydrate was dissolved in 5 ml of DMF (for ZnO thin films). A similar procedure was followed for the hybrid thin films, adding measured amounts of the precursors: ytterbium chloride hexahydrate and europium chloride hexahydrate to obtain a precursor concentration of 20% wt. The resulting solutions were stirred on a magnetic stirrer for 24 h, and then the precursors/DMF were combined with PVP/EtOH and stirred for another 24 h.

**Figure 1:**
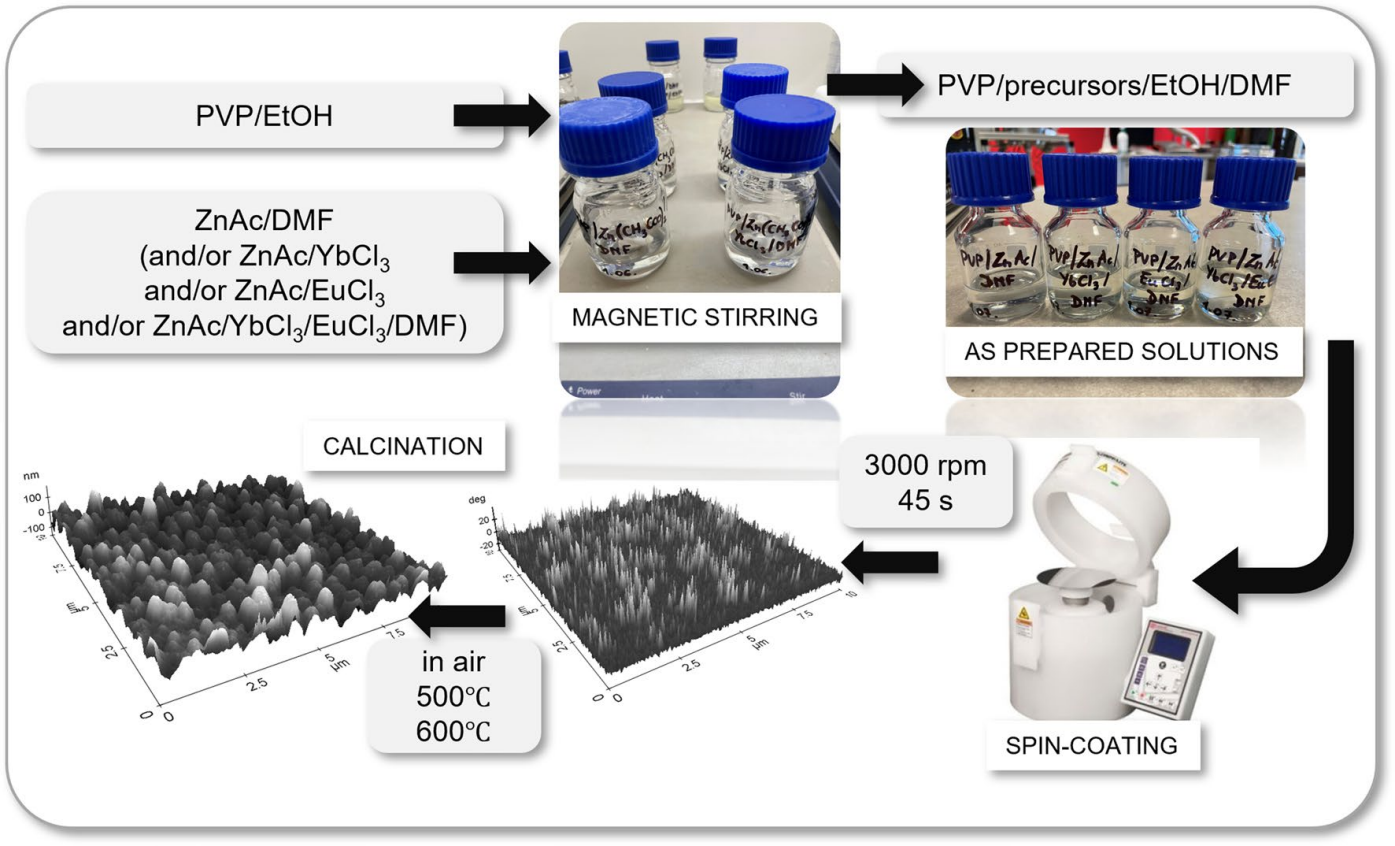
Scheme presenting the methodology of preparing hybrid thin films.

The next step of the experiment was a spin-coating process (Laurell, model WS-650Mz-23NPPB), which was carried out at room temperature using a rotational speed of 3000 rpm and a coating time of 45 s. Thin films were applied to silicon slides (Fig. 1).

In order to obtain ceramic hybrid thin films, the deposited coatings were calcined at 500 and 600 °C for 3 h, with a heating rate of 2 °C/min under air atmosphere. Table 1 shows the sample designations that were used in the rest of the experiment.

**Table 1:** Sample designations of all prepared thin films samples.

Calcination	Chemical composition of thin films			
	ZnO	ZnO/Yb_2_O_3_	ZnO/Eu_2_O_3_	ZnO/Yb_2_O_3_/Eu_2_O_3_
500 °C	Z_500	ZY_500	ZE_500	ZYE_500
600 °C	Z_600	ZY_600	ZE_600	ZYE_600

The hybrid ceramic thin films obtained by the procedure described above were subjected to tests using advanced testing techniques. The morphology, chemical composition and thickness of doped and undoped ZnO thin films were investigated using a scanning electron microscope (SEM, ZEISS Supra 35) and an energy dispersive X-ray detector (EDX, EDAX Trident XM4). In addition, the topography and roughness of the thin films were examined using an atomic force microscope (AFM, XE-100, Park Systems). Wettability properties were determined using the water sitting droplet method (0.4 µm distilled water). Transmittance and absorption spectra were plotted using a UV–Vis spectrophotometer in the wavelength range of 300–800 nm (Thermo Scientific, Evolution 220). The photocatalytic activity of the hybrid thin films was analyzed based on the decomposition kinetics of 0.025 mM aqueous solution of rhodamine B (RhB/H_2_O), in which the thin film samples were immersed, under solar irradiation using a SOLAR SIMULATOR MODEL #SS150AA artificial solar radiation generator equipped with a 1000 W xenon lamp (AM1.5G). Rhodamine B is a dye from the rhodamine group and is used most commonly as a fluorescent dye, but also for dyeing fabrics. RhB is very soluble in water and its concentration in solutions is directly proportional to the absorption at the absorption maximum of 555 nm, hence its solutions are used to study photocatalytic activity by examining the distribution of the dye over time.

## Results and discussion

### Cross-section, topological and chemical composition examination using scanning electron microscope

**Figure 2:**
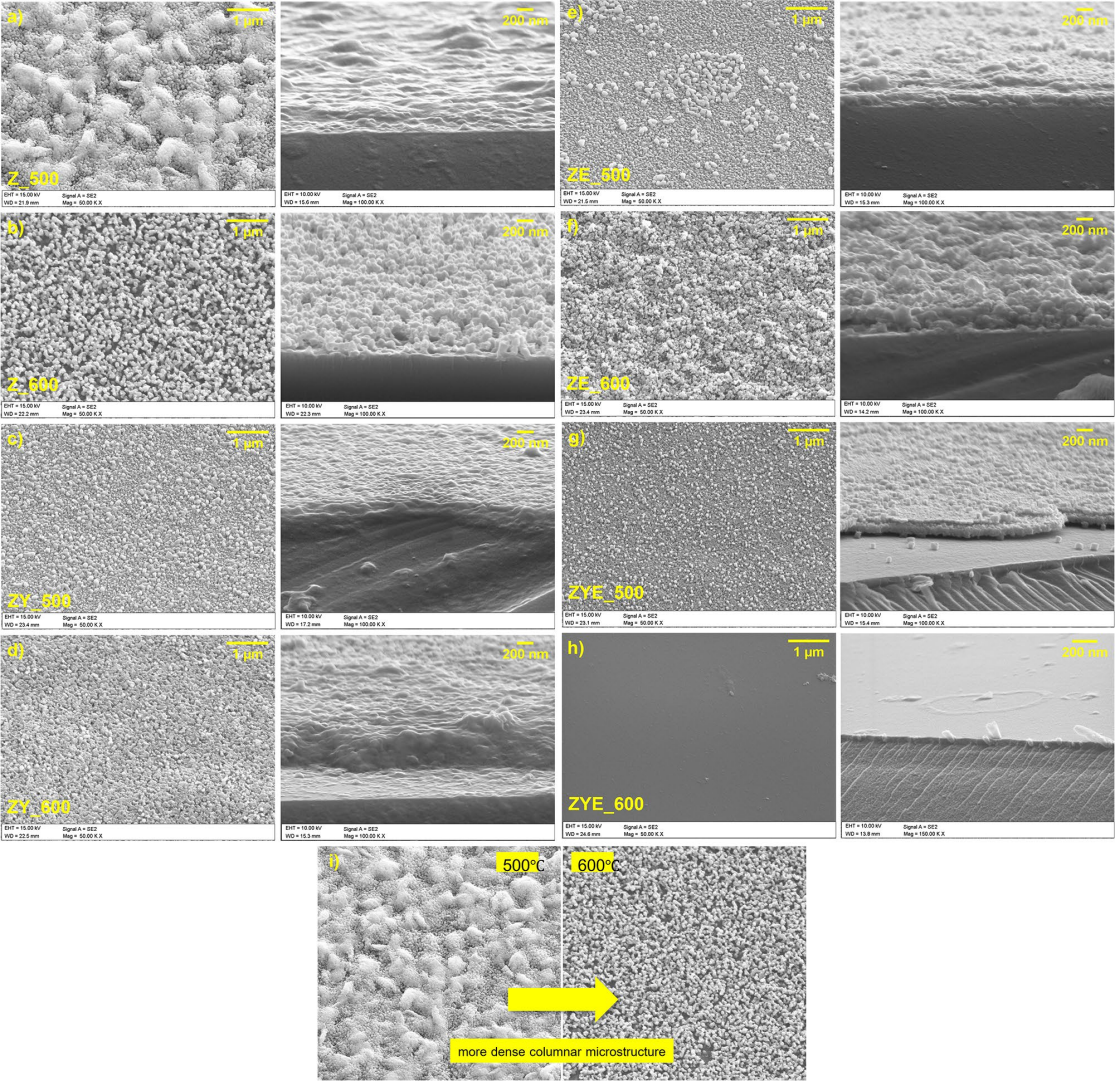
SEM images of hybrid thin films: topography and cross-section (**a**–**h**) and comparison of columnar structure of thin films calcined in two different temperatures.

The topology, chemical composition and approximate thickness values of ZnO and hybrid thin films were investigated using scanning electron microscope (SEM) and energy dispersive spectroscopy (EDS). Figure 2 shows SEM images of thin film topography and cross-section of thin film samples.

Based on SEM images, the structure of spin-coated thin films were characterized by different morphology for different ZnO dopants and calcination temperature. All coatings samples are free of microcracks and other structural defects. Moreover, it can be observed that with higher calcination temperature, the structure of thin films are denser, that in the case of coatings calcinated at 500 °C. It is also observed that metal oxide grains are randomly distributed throughout all the samples, and in the case of pure ZnO thin films, the average grain size increased with the increased calcination temperature. The opposite observation was made based on SEM images of ytterbium and europium oxides—doped ZnO coatings, with the increase in calcination temperature, the average grain size decrease. The agglomeration of grains in ZE_500 coating, which are much higher than the other grains in the thin film structure can be attributed to the europium oxide grains. Zinc oxide thin films structure doped with ytterbium and europium oxide showed the smallest grain size.

Fracture analysis of thin film samples allowed to determine approximate thickness values of undoped ZnO and doped ZnO thin films, whose values ranged from 25 to more than 400 nm. Based on the thickness analysis of pure ZnO films and hybrid ZnO films doped simultaneously with Yb_2_O_3_ and Eu_2_O_3_, it was noted that doping with rare earth oxides resulted in an increase in the thickness of the coatings, a condition believed to be due to competing thickening processes of the thin films occurring before, during and after coalescence, which affect the grain size. The researchers noted that an increase in coating thickness occurs when coalescence is taking place through the exchange of material by atomic self-diffusion between atoms of thin films on the film surface, but also by diffusion along and across grain boundaries to reduce grain boundary density with greater energy. The increase in film thickness at higher temperatures is related to film thickening and grain growth processes, which occur faster at higher temperatures^51^. In addition, the crystal lattice energy rises with increasing atomic number of elements, thus, ytterbium oxide has the second highest crystal lattice energy value among the lanthanides, which results in agglomeration and growth of crystallites at higher temperatures, resulting in increased thickness and roughness of thin films^52^.

**Figure 3:**
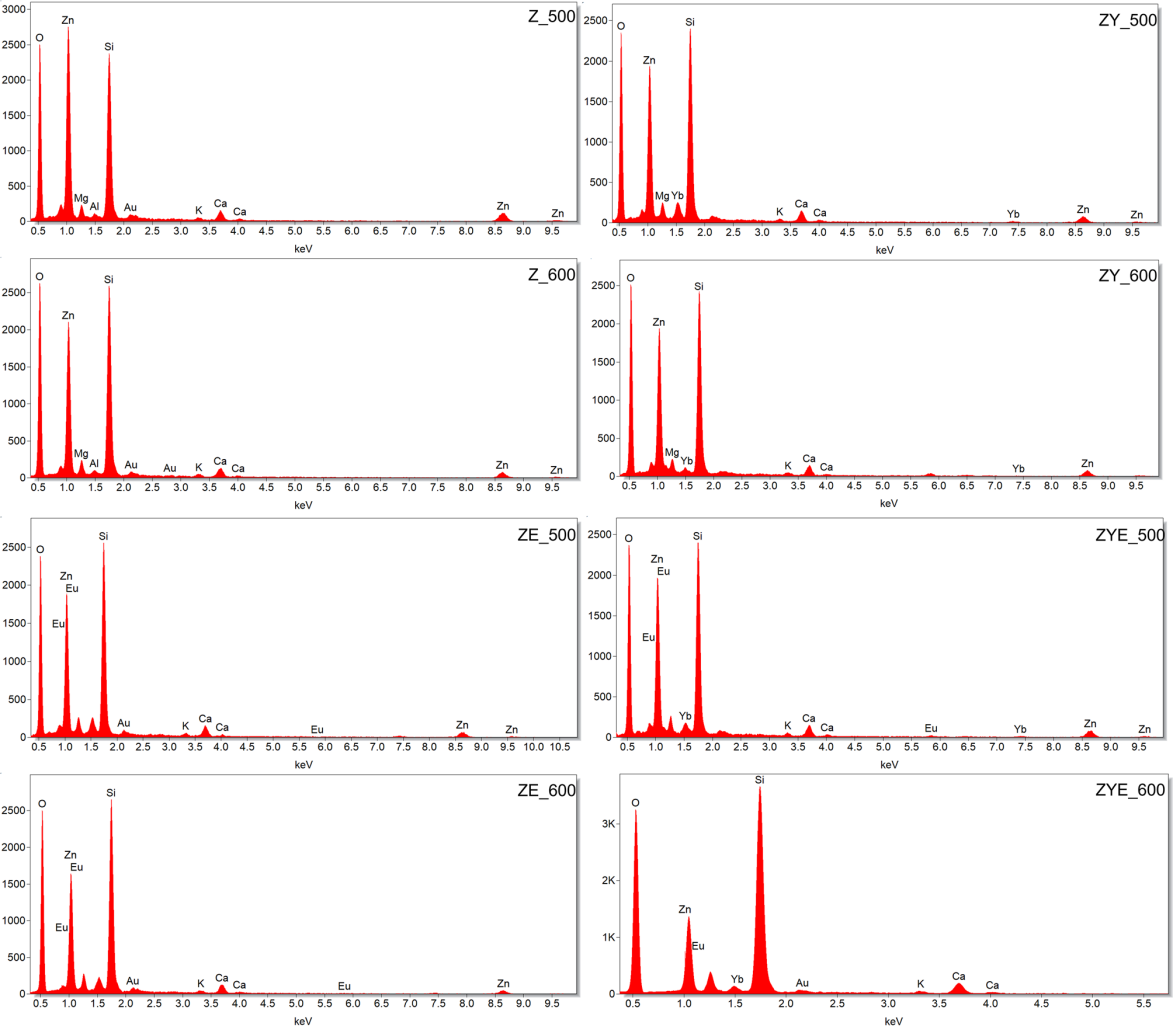
EDS analysis of ceramic hybrid thin films calcined at 500 and 600 ℃.

The chemical composition of the thin films was confirmed by EDS analysis, which showed the presence of elements characteristic for the spin-coating sol–gel thin films: zinc (Zn), oxygen (O), ytterbium (Yb) and europium (Eu). The presence of the elements silicon (Si), magnesium (Mg), calcium (Ca), aluminum (Al), and potassium (K) is responsible for the glass substrate onto which the ZnO and hybrid thin films were deposited (Fig. 3). The samples were sputtered with a thin layer of gold (Au) to be able to dissipate the electrical charge, for a better characterization of the preparations and a clear image of their surface.

### Topological and surface roughness analysis

**Figure 4:**
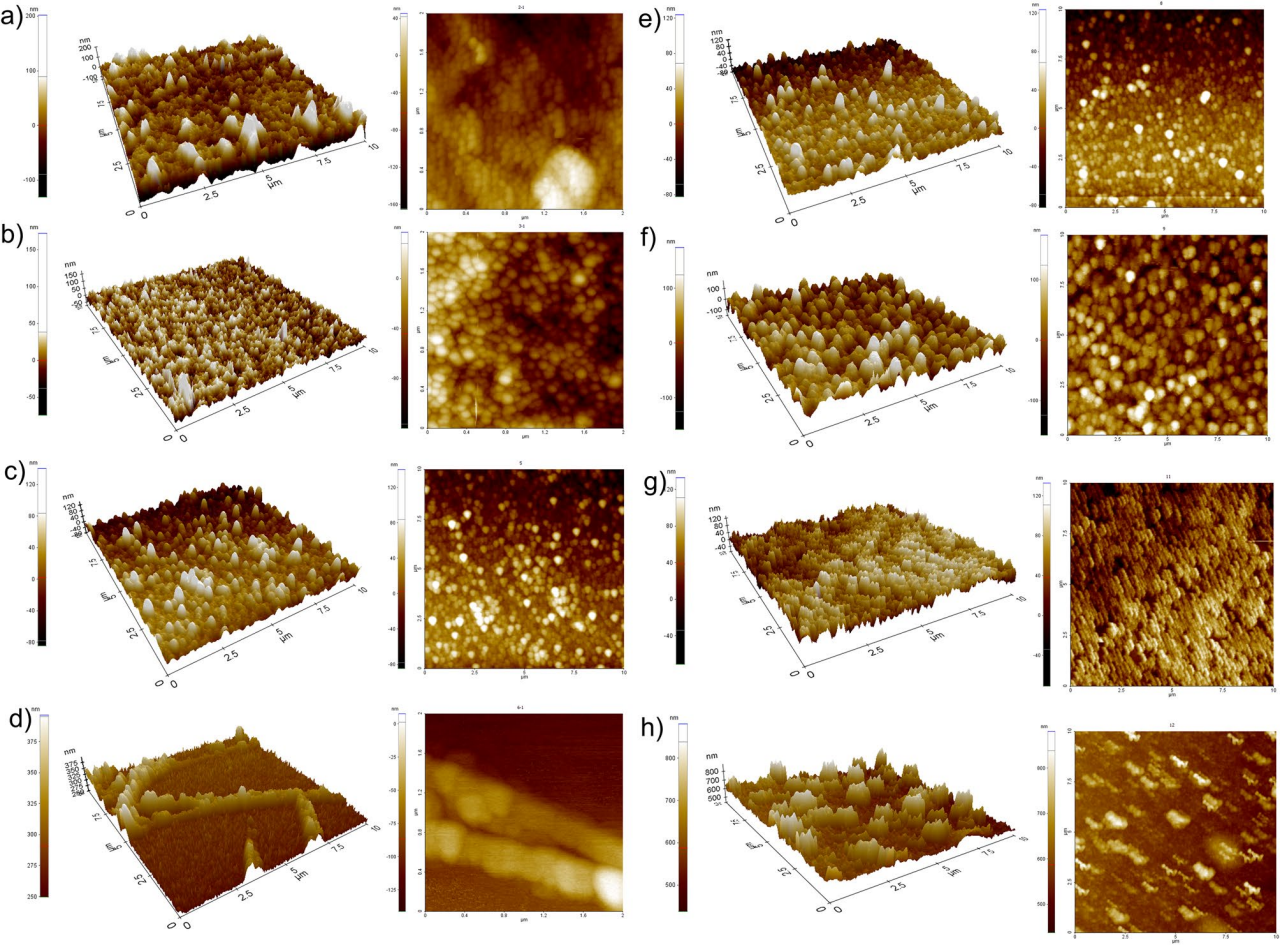
A 3D and 2D AFM images of scanned micro-areas of 10 × 10 µm thin films: (**a**) Z_500, (**b**) Z_600, (**c**) ZY_500, (**d**) ZY_600, (**e**) ZE_500, (**f**) ZE_600, (**g**) ZYE_500 and (**h**) ZYE_600.

**Figure 5:**
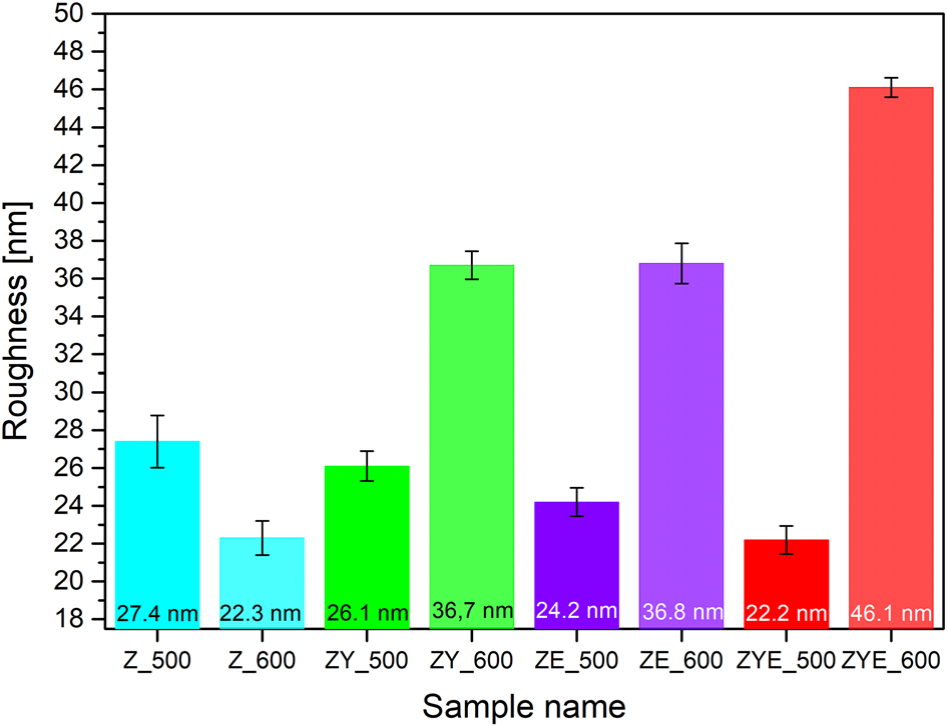
Roughness values (R_a_) of thin films samples in nanometers (with error bars).

In order to determine the relationship of roughness, surface topology and wettability of the hybrid thin films, an atomic force microscope (AFM) study was performed. Based on the obtained images of scanned micro-areas of 10 × 10 µm thin films, it was shown that all thin film samples were characterized by polycrystalline structure with specific roughness (Fig. 4a–h). The procedure of doping ZnO thin films with rare earth oxides and then calcined at 500 °C caused a decrease in roughness in the range of 27.4 to 22.2 nm according to the order of samples Z_500 > ZY_500 > ZE_500 > ZYE_500, which may be related to the segregation of rare earth metals into non-crystalline regions of grain boundaries^53^ (Fig. 5). In addition, the increase in calcination temperature caused an increase in roughness with the doping treatment of ZnO thin films, which is due to the fact of significant grain growth at higher temperature and was also confirmed by scanning electron microscopy studies^47,54,55^.

In both SEM and AFM images of hybrid thin films calcined at 500 °C, it can be observed that the addition of rare earth oxides resulted in a conical columnar microstructure of crystallites, which is mediated by the preferential growth of crystallites along the *c*-axis perpendicular to the substrate, resulting in a decrease in roughness and differences in height. Despite the lowest thickness of the ZYE_600 thin film determined from the scanning electron microscope image of the sample breakthrough, the presence of rare earth oxide crystallites significantly increased the coating roughness (Fig. 2). The high roughness suggests a more efficient scattering of incident light on the layers, which will result in an increase in the optical path length of radiation inside, for example, photovoltaic cells. An undesirable effect caused by too high roughness values (above 150 nm) is scattering of radiation at too large angles and secondary absorption by the coating. The obtained results demonstrate the potential application of hybrid self-cleaning thin films on photovoltaic panels^56^.

### Water contact angle measurements and wettability examination

**Figure 6:**
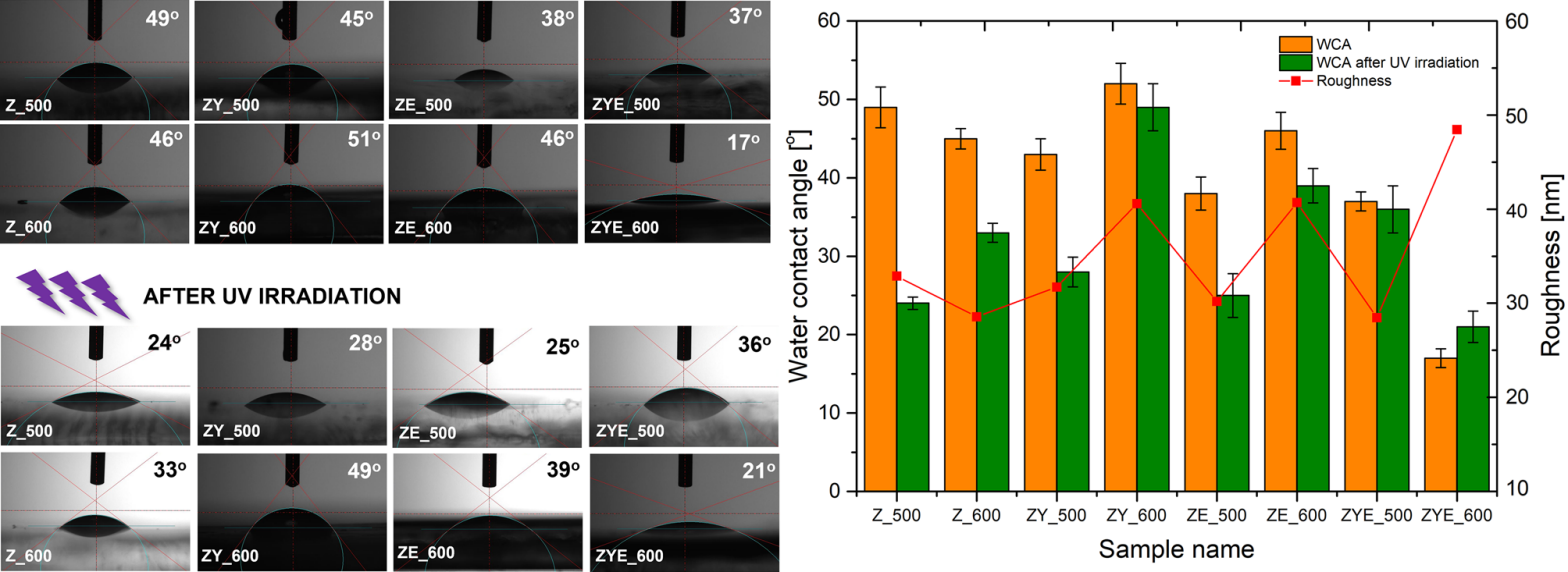
Water contact angle measurements of thin films: before and after UV light irradiation and scheme presenting the dependence of water contact angle values before and after UV irradiation on roughness of thin films.

Wettability of self-cleaning coatings is a key issue determining the cleaning mechanism and, in the case of photocatalytically active thin films, additionally the efficiency of the chemical removal process of dust and dirt particles by the coating. In order to determine the water contact angles of the obtained thin films, three measurements of each analyzed sample were performed using the 0.4 µm sitting water droplet method (and the average value was calculated). Furthermore, in order to determine the effect of solar radiation on wettability, the thin films were irradiated with ultraviolet radiation (λ = 365 nm) for a period of 30 min and then the wettability angle was measured three times again. All measurements were performed at ambient temperature. Photographs of the water droplets on the surface of the thin films and the results of the wetting angle measurements are shown in Fig. 6.

The obtained results of water angle measurements for all thin film samples indicate the hydrophilic nature of the surface, showing wetting angle values between 17 and 51°. Moreover, for the pure ZnO and ZnO/Yb_2_O_3_/Eu_2_O_3_ thin film samples, the wetting angle values were also reduced with the increase of calcination temperature, with the largest decrease in the values observed for the ZYE_600 sample, which simultaneously has the highest roughness and the lowest film thickness (Fig. 6). Subjecting ZnO and ZnO thin films doped with rare earth oxides to UV irradiation affected the wetting angle values^57^. The noticeable decrease in the wetting angles and thus in the increase in wettability after UV exposure is related to the interaction of electron holes with oxygen released from the crystal lattice of oxide materials and, as a result, to the formation of oxygen gaps. As a consequence, the binding energy of the metal to the oxygen atom in the oxide layer is reduced and the oxygen vacancies induce the adsorption of hydroxyl groups (–OH), as a result of which the hydrophilicity of the thin film surface increases. The mechanism of variable wettability of oxide surfaces is related to the absorption of photons having energy equal to or greater than the energy of the energy gap, under the influence of which the excited electron migrates to the conduction band, leaving an electron hole in the valence band. The photoinduced electrons and electron holes recombine or migrate to the surface of the film to react with impurities adsorbed on the surface of the thin film. The resulting electron holes in the valence band react with water molecules adsorbed on the film surface to form hydroxyl free radicals (OH*), and electrons in the conduction band reduce oxygen molecules to superoxide anions (O_2_*). OH* and O_2_* are extremely reactive and exhibit strong oxidizing properties, thus participating in the degradation of compounds present on the surface of oxide coatings^25,58^. It is also worth mentioning that increasing the calcination temperature to 600 °C resulted in an increase in the wetting angle values compared to the θ values of thin films calcined at a lower temperature.

Figure 6 shows the dependence of wettability on surface roughness of thin films. All the samples calcined at 500 °C show a trend of reduction in wetting angle value with decrease in roughness in the order Z_500 > ZY_500 > ZE_500 > ZYE_500. This is due to the doping process of the ZnO thin films, which in the order shown, are characterized by increased heterogeneity, as well as a more compact microstructure (Figs. 2, 3a,c,e,g), in effect presenting a roughness-wettability relationship characteristic of the Cassie—Baxter model^59^. Thin films calcined at 600 °C, which show an increase in surface roughness with increasing microstructural heterogeneity, are instead characterized by the Cassie–Baxter–Wenzel mixed model. In this model, air "pockets" located in the columnar microstructure of the surface of thin films cease to be thermodynamically stable and undergo wetting, so that in samples Z_600, ZY_600 and ZE_600 no dependence of the increase in wettability (decrease in the value of the wetting angle) on the increase in roughness was observed. The only exception to this rule is the ZnO/Yb_2_O_3_/Eu_2_O_3_ sample calcined at a higher temperature, which shows the highest surface roughness value among all thin films produced by spin-coating, while having the lowest wetting angle values and thus the best wettability of all samples. This phenomenon is probably related to the condition of the so-called surface film (pseudo-Wenzel model) for surfaces for which the Wenzel model does not apply, and the droplet wetting the coating surface extends its penetration front beyond the droplet boundaries and forms a liquid layer on the thin film surface^60^. This sample also exhibits the least uniform microstructure of all thin films, providing a high wettability of the film that is not reduced by the presence of rare earth oxide crystallites (Fig. 2). In conclusion, the obtained ZnO and ZnO thin films doped with rare earth oxides exhibit hydrophilic wettability properties, and UV irradiation deepens their hydrophilicity, thus increasing the wettability of the films. In the case of self-cleaning coatings used for photovoltaic panels, among others, the high wettability of the coatings plays an extremely important role, defining the hydrophilic mechanism of the surface cleaning process.

### Optical properties of pure and RE oxides doped ZnO thin films

**Figure 7:**
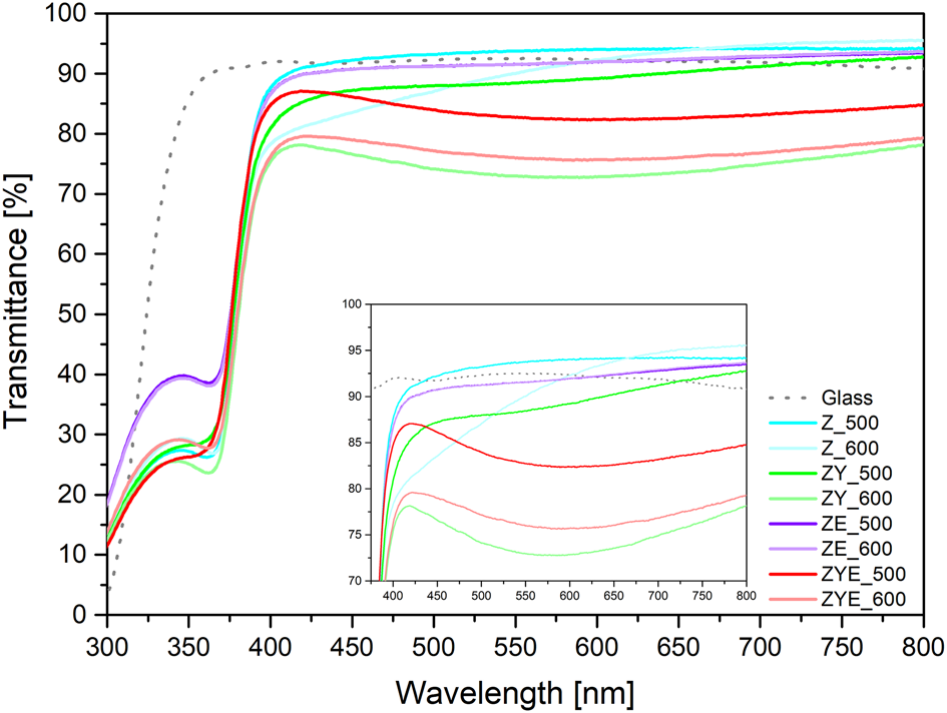
Transmittance spectra of ZnO and rare earth metal oxide-doped ZnO thin films.

Figure 7 shows the transmittance plots as a function of electromagnetic radiation wavelength 300–800 nm of hybrid calcined thin films at two temperatures (500 and 600 °C) obtained using UV–Vis spectrophotometer.

From Fig. 7, it can be observed that all the hybrid thin films have high optical transmittance in the visible light range exceeding 73%, and the cut-off wavelength value is for about 380 nm^43^. The transmittance of all analyzed thin film samples ranges from 73%, for sample ZY_600 to a value of 93.7% for sample Z_500 (values measured at 550 nm) (Table 2).

**Table 2:** Optical transmittance of hybrid thin films (for λ = 550 nm).

Calcination temperature	Optical transmittance (%)			
	ZnO	ZnO/Yb_2_O_3_	ZnO/Eu_2_O_3_	ZnO/Yb_2_O_3_/Eu_2_O_3_
500 °C	93.7	88.7	91.5	82.9
600 °C	90.6	72.9	91.5	76

Moreover, hybrid ZnO thin films doped with Eu_2_O_3_ showed a higher transmittance in the near ultraviolet range, compared to ZnO, ZnO/Yb_2_O_3_ and ZnO/Yb_2_O_3_/Eu_2_O_3_ thin films. This fact is probably related to the overlapping cut-off wavelengths in the transmittance diagram of both oxides: zinc oxide and europium oxide^61^. Moreover, the lack of noticeable signal coming from Yb_2_O_3_ in the sample of ZY_500 and ZY_600 thin films is caused by the overlapping absorption edges of ZnO and Yb_2_O_3_ thin films, for which the absorption maximum falls at ~ 350–360 nm^62^. A noticeable trend in the dependence of the transmittance value on the calcination temperature is that the transmittance decreases with increasing temperature from 500 to 600 °C. This phenomenon is related to the more compact crystallite structure of thin films and crystallite growth at higher temperatures, which was confirmed by topography and roughness analysis performed using atomic force microscopy and topography analysis of SEM images.

For straight energy gap materials such as ZnO, the dependence of the absorption coefficient (α) and energy gap width (Eg) can be expressed by the Tauc formula:

$$(\alpha h\upsilon {)}^{n}=B(h\upsilon -{E}_{g})$$

where α is the absorption coefficient, hυ is the photon energy of the incident radiation on the sample, n is the value defining the oblique or straight energy gap (for straight gap n = 2), B is the optical constant, and Eg is the determined energy gap width (eV).

**Figure 8:**
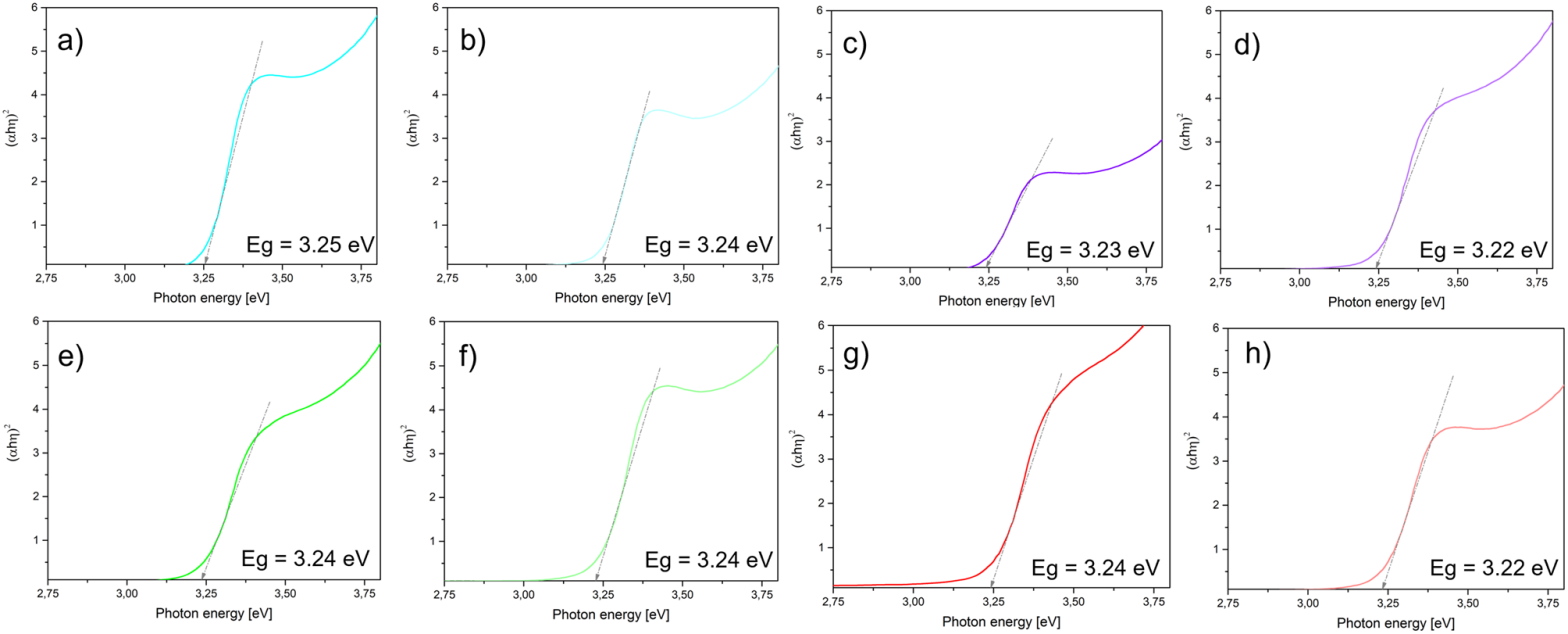
Dependence of $(\alpha h\upsilon {)}^{2}$ as a function of photon energy with a linear relationship for α = 0 and the determined values of the optical energy gap of thin hybrid films.

Figure 8 shows plots of $(\alpha h\upsilon {)}^{2}$ as a function of photon energy and extrapolation of the straight line for α = 0. The energy gap widths of all the thin films obtained range from 3.22 eV to a maximum value of 3.25 eV. The highest Eg value was recorded for the ZnO thin film calcined at 500 °C, and the lowest Eg value was recorded for the ZnO/Yb_2_O_3_/Eu_2_O_3_ hybrid sample calcined at 600 °C. The procedure of doping ZnO with rare earth oxides reduced the energy gap widths of the hybrid coatings. This phenomenon can probably be attributed to the negative Burtsein-Moss effect due to the increased concentration of charge carriers in the rare earth oxide doped ZnO films^63^. Based on the results obtained from the calculation of the widths of the optical gaps of the oxide thin films, the samples calcined at 600 °C, which exhibit the lowest Eg values, the highest surface roughness values, and favorable wettability properties, were selected for the photocatalytic activity studies.

### Photocatalytic activity examination based on rhodamine B degradation

**Figure 9:**
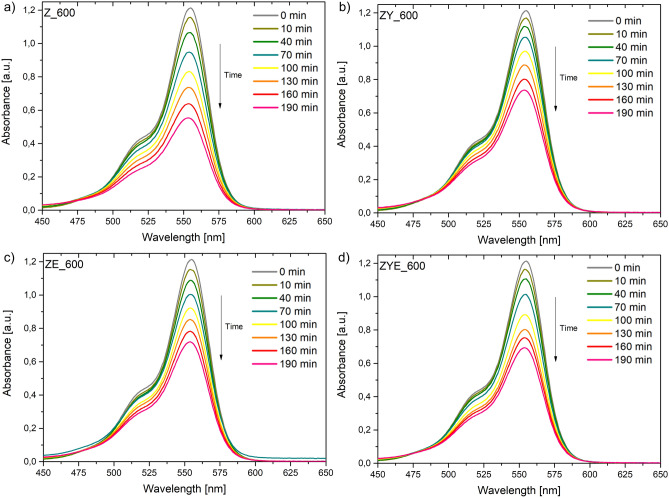
Dependence of absorption as a function of exposure time to solar radiation of the aqueous RhB solution in the presence of a photocatalyst in the form of a thin layer: (**a**) ZnO, (**b**) ZnO/Yb_2_O_3_, (**c**) ZnO/Eu_2_O_3_, (**d**) ZnO/Yb_2_O_3_/Eu_2_O_3_.

In order to investigate the photocatalytic activity of ZnO and ZnO thin films doped with rare earth oxides, the decolorization process of rhodamine B aqueous solution under solar radiation (SOLAR SIMULATOR MODEL #SS150AA) was carried out. The measurement procedure was carried out for 190 min (with the first absorption measurement performed after 10 min and subsequent measurements every 30 min) (Fig. 9). The noticeable decrease in the degree of absorption over time for a wavelength of about 555 nm (absorption maximum of rhodamine B) is related to the decolorization process and the decrease in absorption of the RhB/H_2_O solution under the influence of photocatalytic thin films and solar radiation.

**Figure 10:**
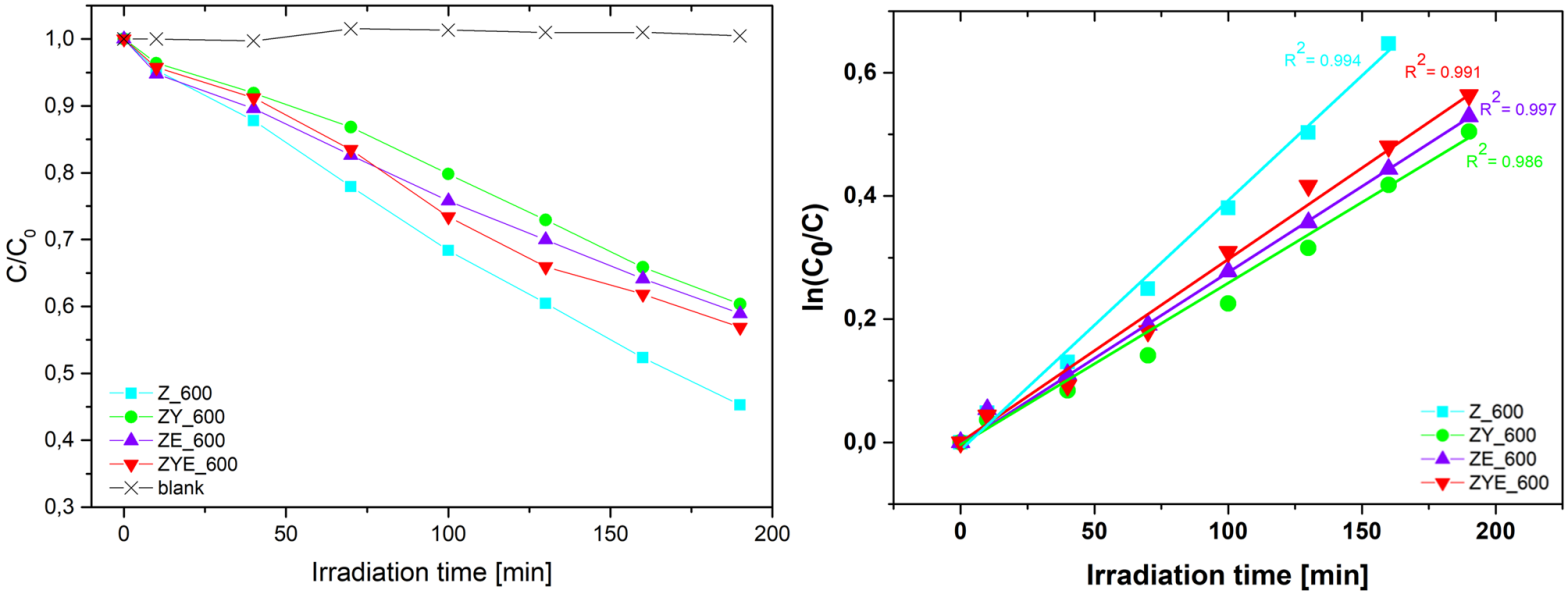
Graph of rhodamine B decolorization as a function of exposure time in the presence of a photocatalyst in the form of thin ZnO and hybrid layers and without the presence of a photocatalyst (blank) and kinetics of rhodamine B decolorization in the presence of a pseudo-first order photocatalyst with linear fit and R^2^ values.

Figure 10 shows the degree of RhB/H_2_O decolorization (C_t_/C_0_) as a function of measurement time. The decolorization of the dye in aqueous solution in the presence of a photocatalyst can be calculated using the formula:

$$\eta =\left[{(C}_{0}-{C}_{t}\right)/{C}_{0}]=[({A}_{0}-{A}_{t})/{A}_{0}]$$

where C_0_ or A_0_ is the initial concentration or absorption of RhB (at an absorption maximum of λ = 555 nm), respectively, and C_t_ or A_t_ is the concentration or absorption at a given measurement time during the photocatalysis process (from 10 to 190 min).

From Fig. 10, it can be observed that the highest photocatalytic activity at 190 min in RhB aqueous solution is characterized by sample Z_600, which degraded 54.7% of the dye at 190 min^64^, and the lowest by ZY_600 thin films, which have a degradation rate of 39.6% under solar irradiation. For ZnO thin films doped with europium oxide and ZnO doped with both ytterbium oxide and europium oxide, the degradation rates were, respectively: 41,1% and 43,1%. Based on the above, the order of thin film samples in terms of their photocatalytic activity under solar radiation is as follows: Z_600 > ZYE_600 > ZE_600 > ZY_600. It is further worth noting that doping with rare earth oxides: Yb_2_O_3_ or Eu_2_O_3_ did not significantly improve the performance of RhB photodegradation process compared to undoped ZnO thin film. However, the combination of both rare earth oxides as dopants of the ZnO film represents the resultant C/C_0_ dependence of the ZnO thin film and the doped layers of each REM oxide separately. The lower photocatalytic activity of the ZYE_600 thin film compared to Z_600 may be due to the much lower thickness, less than 50 nm, compared to the ~ 90 nm thickness of the Z_600 film. When the layer is thinner only a small amount of radiation is absorbed by the nanophotocatalyst^65^. Nevertheless, the use of doping in the form of both oxides: ytterbium and europium ZnO thin films improves the photocatalytic properties of the coating compared to doping each oxide separately.

The kinetics of the decolorization reaction of an aqueous solution of rhodamine B in the presence of thin film photocatalysts are shown in Fig. 10. In order to determine and fit the corresponding kinetics, the ln(C_0_/C) dependence as a function of the decolorization process time was plotted, from which linear regression was then performed and the corresponding order values of the reaction kinetics were fitted. ZnO and ZnO thin films doped with rare earth oxides exhibited pseudo-first-order characteristic decolorization reaction kinetics, with R^2^ of 0.994, 0.986, 0.997, and 0.991 for Z_600, ZY_600, ZE_600, and ZYE_600, respectively. Furthermore, the reaction constants were determined from the determined linear functions of the decolorization reaction kinetics, which were as follows: 4.11 × 10^–3^ min^−1^, 2.62 × 10^–3^ min^−1^, 2.72 × 10^–3^ min^−1^ and 3.04 × 10^–3^ min^−1^ for ZnO, ZnO/Yb_2_O_3_, ZnO/Eu_2_O_3_ and ZnO/Yb_2_O_3_/Eu_2_O_3_ thin films, respectively. According to other works, reaction kinetics for ZnO are in good agreement with^66,67^. The photocatalytic activity results correspond with those reported in other research works in the area of semiconductor thin films (Table 3).

**Table 3:** Comparison between different ZnO thin films efficiencies in other works.

Production method of ZnO thin film	Dye/irradiation type	Time (min)	Efficiency (%)	Kinetics (min^−1^)	Ref.
Sol–gel (spin-coating)	RhB/sunlight	240	93	NS	^68^
DC sputtering and pulsed laser deposition (PLD)	RhB/sunlight	360	60.85	NS	^69^
DS reactive sputtering	RhB/sunlight	180	38	NS	^70^
Sol–gel (dip-coating)	RhB/UV light	240	66	NS	^64^
Sol–gel (spin-coating)	RhB/UV light	240	50	NS	^71^
Sol–gel (spin-coating)	RhB/UV light	120	64	NS	^72^
Sol–gel (spin-coating)	RhB/UV light	240	32–77	1.6–6.2 × 10^–3^	^73^
Spray pyrolysis	RhB/sunlight	120	38	2.7 × 10^–3^	^74^
Sol–gel (spin-coating)	RhB/sunlight	190	54.7	4.11 × 10^–3^	This work

*NS* not studied.

The obtained results of the efficiency of photocatalytic activity of thin films produced by sol–gel technique in combination with spin-coating, together with the roughness values, wettability characteristics of the coatings testify to the potential application successfully as self-cleaning coatings for photovoltaic panels and glass structures in architecture.

## Conclusion

This work presents an economical method for the fabrication of hydrophilic ZnO and ZnO thin films doped with rare earth metals that are photocatalytically active and can be used as self-cleaning coatings for photovoltaic panels or glass structures. The microcolumnar polycrystalline structure of the hydrophilic thin films was characterized by roughness values not exceeding 50 nm, thus providing efficient absorption of radiation inside the structure. Moreover, a decrease in the wetting angle values caused by ultraviolet irradiation was observed, indicating the switchable nature of the wettability of the obtained coatings. The high optical transmittance, exceeding 76%, in the visible light region provides very low radiant energy losses assuming the use of the hybrid thin films as coatings for photovoltaic panels. Furthermore, the self-cleaning properties of the coatings, in addition to mechanical cleaning of dirt and dust using water droplets, were demonstrated by photocatalytic activity studies, resulting in a range of values for the decolorization rate kinetics of rhodamine B solution in the range of 2.62 × 10^–3^ min^−1^ to 4.11 × 10^–3^ min^−1^, which provides an additional chemical cleaning mechanism. Thus, the combination of the sol–gel technique and the spin-coating method allows the fabrication of self-cleaning coatings according to a hydrophilic mechanism combined with chemical photocatalytic removal of contaminants under solar radiation, which can be successfully applied to photovoltaic panels or architectural glass structures eliminating the need for manual cleaning.
